# Supplementary material for: Chondroitin 6-sulfate represses keratinocyte proliferation in mouse skin, which is associated with psoriasis
Source: Commun Biol. 2021 Jan 25;4:114. doi: 10.1038/s42003-020-01618-5 (PMC7835381; doi:10.1038/s42003-020-01618-5)
Supplement: Supplementary file 2 — Supplementary Information [file 42003_2020_1618_MOESM2_ESM.pdf]

Supplementary Figure 1

Figure 3c

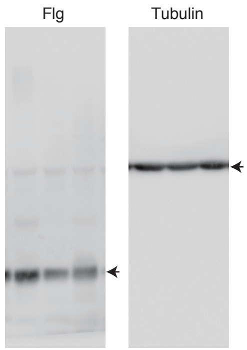

Figure 4a

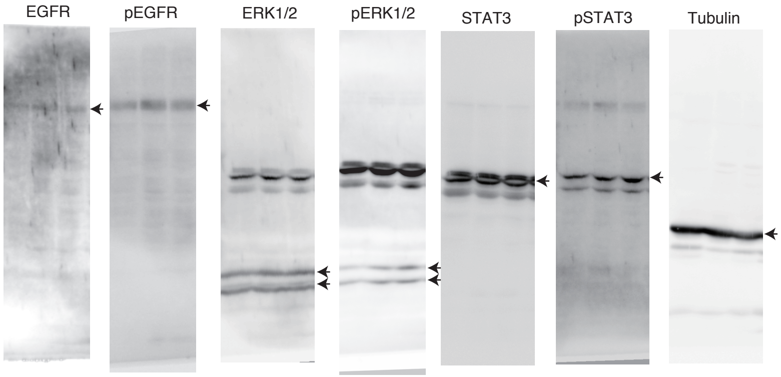

Figure 5b

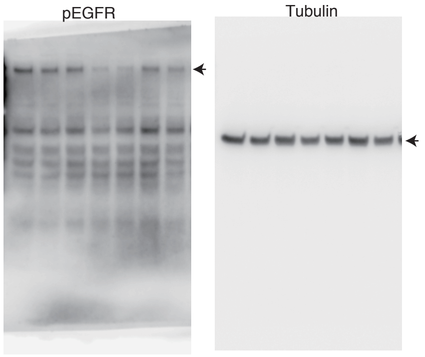

Figure 5c

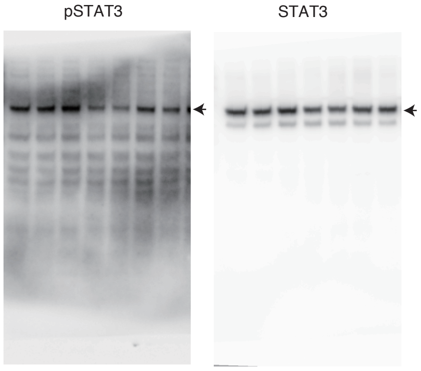

Figure 7b

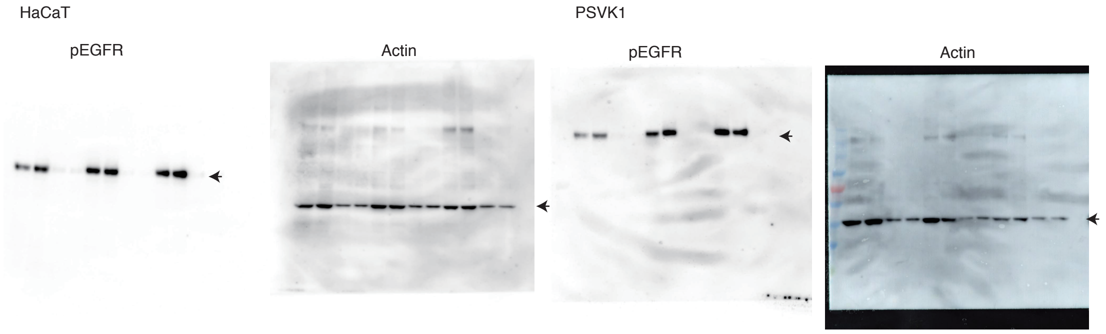

Figure 8h

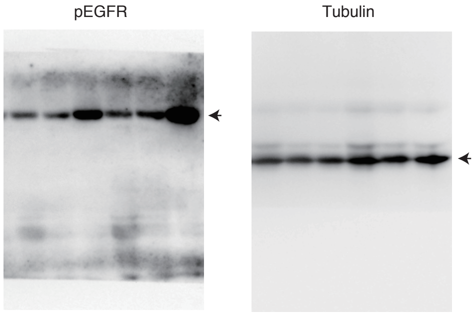

Supplementary Table 1

Disaccharide composition of CS chains from skin of WT (*C6st-1*$^{+/+}$), HET (*C6st-1*$^{+/-}$), and KO (*C6st-1*$^{-/-}$) newborn mice.

Values are expressed as a percent of total disaccharide units, and the means ± s.d. of three determinations.

CS-disaccharide unit	CS-disaccharides (mol %)		
	WT	HET	KO
ΔDi-0S[a]	22.6±0.7	18.9±4.9	21.0±2.4
ΔDi-6S	11.4±3.7	4.2±1.3	ND
ΔDi-4S	61.4±4.4	71.6±3.4	75.1±1.9
ΔDi-diS$_D$	1.0±0.2	ND	ND
ΔDi-diS$_E$	3.5±1.1	5.3±2.1	4.0±3.0

[a]Abbreviations:

ΔDi-0S	ΔHexAα1-3GalNAc
ΔDi-6S	ΔHexAα1-3GalNAc(6-*O*-Sulfate)
ΔDi-4S	ΔHexAα1-3GalNAc(4-*O*-Sulfate)
ΔDi-diS$_D$	ΔHexA(2-*O*-Sulfate)α1-3GalNAc(6-*O*-Sulfate)
ΔDi-diS$_E$	ΔHexAα1-3GalNAc(4-, 6-*O*-Sulfate))

[b]ND, not detected.

Supplementary Table 2

Disaccharide composition of CS chains from primary mouse keratinocytes and two human keratinocyte cell lines, HaCaT and PSVK1.

Values are expressed as pmol of disaccharide per mg of dried homogenates of these cells, and the means ± s.d. of three determinations.

	Primary mouse keratinocytes	HaCaT	PSVK1
CS disaccharides (pmol/mg) (mol %)			
ΔDi-0S[a]	8.1±2.4 (24.0)	5.1±0.8 (1.9)	3.3±2.4 (6.8)
ΔDi-6S	2.4±0.2 (7.1)	27.9±3.9 (10.3)	11.5±0.5 (23.9)
ΔDi-4S	20.1±4.1 (59.5)	231.2±42.2 (85.6)	28.0±9.7 (58.0)
ΔDi-diS$_D$	1.1±0.2 (3.3)	2.4±1.1 (0.9)	0.3±0.1 (0.7)
ΔDi-diS$_E$	2.1±0.4 (6.1)	3.5±4.1 (1.3)	5.1±1.5 (10.6)
ΔDi-triS	ND[b]	ND	ND
Total amount (pmol/mg)			
	33.8±7.0	270.2±50.4	48.3±11.9

Values are expressed as pmol of disaccharide per mg of dried cell homogenates and as means ± s.d. of three determinations.

[a]Abbreviations: ΔDi-0S, ΔHexAα1-3GalNAc; ΔDi-6S, ΔHexAα1-3GalNAc(6-*O*-Sulfate) ; ΔDi-4S, ΔHexAα1-3GalNAc(4-*O*-Sulfate); ΔDi-diS$_D$, ΔHexA(2-*O*-Sulfate)α1-3GalNAc(6-*O*-Sulfate); ΔDi-diS$_E$, ΔHexAα1-3GalNAc(4-, 6-*O*-Sulfate)); ΔDi-triS, ΔHexA(2-*O*-Sulfate)α1-3GalNAc(4-, 6-*O*-Sulfate);

[b]ND, not detected.

Supplementary Table 3

Primers used for real-time PCR

Gene Name	5'-primer	3'-primer
mAREG	5'-GGTCTTAGGCTCAGGCCATTA-3'	5'-CGCTTATGGTGGAAACCTCTC-3'
mCLDN1	5'-TATCCCTTACAGATCGCTGGAC-3'	5'-CTGCCGATGAAAGCTGACACT-3'
mCLDN5	5'-GCAAGGTGTATGAATCTGTGCT-3'	5'-GTCAAGGTAACAAAGAGTGCCA-3'
mEGF	5'-AGCATCTCTCGGATTGACCCA-3'	5'-CCTGTCCCGTTAAGGAAAACTCT-3'
mFAM20B	5'-GCCTTAAAATCTGCCATGGC-3'	5'-GGCATCCTGTCTTCCACCAG-3'
mG3PDH	5'-CATCTGAGGGCCCACTG-3'	5'-GAGGCCATGTAGGCCATGA-3'
mHB-EGF	5'-CGGGGAGTGCAGATACCTG-3'	5'-TTCTCCACTGGTAGAGTCAGC-3'
mIL-1β	5'-GATCCACACTCTCCAGCTGCA-3'	5'-GATCCACACTCTCCAGCTGCA-3'
mIL-6	5'-TAGTCCTTCCTACCCCAATTTCC-3'	5'-TTGGTCCTTAGCCACTCCTTC-3'
mIL-17a	5'-CCACGTCACCCTGGACTCTC-3'	5'-CTCCGCATTGACACAGCG-3'
mIL-23a	5'-ATGCTGGATTGCAGAGCAGTA-3'	5'-ACGGGGCACATTATTTTTAGTCT-3'
mIvl	5'-GTCCCATCAACACACACTGC-3'	5'-CTCCTCATGTTTGGGAAAGC-3'
mK10	5'-GCCTCCTACATGGACAAAGTC-3'	5'-GCTTCTCGTACCACTCCTTGA-3'
mK14	5'-CAGTATCCGATCTCTTCATGCG-3'	5'-GGGCTCACAGCCGGTTTCCTG-3'
mLOR	5'-GCGGATCGTCCCAACAGTATC-3'	5'-TGAGAGGAGTAATAGCCCCCT-3'
mOCLN	5'-TTGAAAGTCCACCTCCTTACAGA-3'	5'-CCGGATAAAAAGAGTACGCTGG-3'
mTGF-α	5'-CACTCTGGGTACGTGGGTG-3'	5'-CACAGGTGATAATGAGGACAGC-3'
mTMG1	5'-TCTGGGCTCGTTGTTGTGG-3'	5'-AACCAGCATTCCCTCTCGGA-3'
mTNF-α	5'-CATCTTCTCAAAATTCGAGTGACAA-3'	5'-TGGGAGTAGACAAGGTACAACCC-3'
