# Supplementary material for: Chondroitin 6-sulfate represses keratinocyte proliferation in mouse skin, which is associated with psoriasis
Source: Commun Biol. 2021 Jan 25;4:114. doi: 10.1038/s42003-020-01618-5 (PMC7835381; doi:10.1038/s42003-020-01618-5)
Supplement: Supplementary file 3 — Description of Additional Supplementary File [file 42003_2020_1618_MOESM3_ESM.pdf]

Description of Additional Supplementary File

File name: Supplementary Data 1

Description: Source data underlying the main figures.
